# Who Is Insane?

**Published:** 1916-04-15

**Authors:** Charles Arthur Mercier


### WHO IS INSANE?

By CHARLES ARTHUR MERCIER, M.D.Lond., F.R.C.P.

This is the title of a book recently published by the Macmillan Company at New York, and written by Dr. Stephen Smith, a Doctor both of Medicine and of Laws, who was for six years Commissioner in Lunacy of the State of New York. The chief lesson conveyed by the book is that a man may be a Doctor of Medicine and of Laws, and for six years a Commissioner in Lunacy, and yet know nothing about insanity, and very little about a good many other things. The subject of insanity has a great fascination for those who know nothing about it, and the general interest in it is shown by the large number of anecdotes about insane persons that are in circulation, anecdotes which anyone who knows anything of insanity sees at once to be fictions, since anyone with experience of the insane knows that they are impossible. Yet it is of anecdotes of this character that this book is largely composed.

The question which is taken as a title for the book is an important one, and is at the present time undergoing revision. The answer given to it by both legal and medical authorities has until lately been unanimous, and has been, "He whose mind is unsound." The Lunacy Act, 1890, instructs the certifying doctor to state in his certificate that the subject of it is a lunatic, an idiot, or a person of unsound mind, and it is evident that the framers of the Act regard the latter term as inclusive of the two former, and equivalent to them. The definitions of insanity that are given in medical books are very numerous, and, it may be added, very diverse, but they are all framed in terms of unsoundness of mind. This view, which is sanctioned by universal consent and unanimous practice, has for some years past been challenged by me. I define insanity not in terms of mind, but in terms of conduct or action. According to my view, he is insane, not whose mind is unsound, but whose conduct is unsound: we judge of insanity not by what a person thinks and feels, but by what he says and does; and, moreover, his mind may be greatly disordered without his being in the least insane. This view of insanity is novel, and has consequences of great importance.

### Motive and Conduct.

The new doctrine is based upon the fact, which certainly cannot be questioned, that we cannot observe what another person is thinking or feeling. We cannot see into his mind and watch what is going on there. We can only guess at it, and our guesses are based on observation of what he says and of what he does. We take his behaviour as an index of what is going on in his mind, and judge of the state of his mind by what he says and does; and if by this means we arrive at the conclusion that his mind is unsound we call him insane. If, for instance, he says something which is false and seems extravagant, we say he has a delusion; but this is a mere guess. It may be true, and so-called delusions have often turned out to be true. It may be a mistake, such as any sane person may make; or it may be that the assertor is lying, and is quite well aware of the falseness of his assertion. We may make a shrewd guess at which alternative is the true one, but it can only be a guess. Why not, says the new school, take the assertion itself and judge by that in conjunction with other behaviour of the patient? Supposing a woman in the middle of the night gets out of a window and wanders about the country in her night-dress, with bare feet, and when met and questioned gibbers unintelligibly; is not this enough to prove her insanity? Need we, or do we in fact, investigate the state of her mind before we pronounce her insane? The new school says that we need not, and do not. We judge by her conduct alone. Suppose a man comes to his doctor and says, "My mind is not as clear as I could wish, I get confused over my work, and find I have not the grip of it that I used to have; do you think I had better take a rest? That man has disorder in his mind. It is clearly unsound; but are we to say that he is mad? Does he not prove, by taking the sensible course of consulting his doctor, and proposing the sensible course of taking a rest, that he is not mad?

### Insanity without Delusions.

It is still the common practice of medical men to refuse to certify a patient as insane unless they can find in him evidence of a delusion. This is the direct and inevitable consequence of the doctrine that insanity is disorder of mind, and it is a very disastrous consequence. A young woman is beset by sexual craving. As long as she controls her conduct, and does not give way to her craving, she is not mad; but if she gives way to it, and acts in accordance with it; if she shows herself naked to workmen in the house; if she makes indecent overtures to the footman, the groom, to casual acquaintances, and even to strangers, is she not unquestionably mad, and should she not be confined as a mad woman? The parents of such a woman have for years been trying to get her certified and put under restraint, but they have been met by one doctor after another with the same *non-possumus*, "Your daughter has no delusion, and therefore it is quite impossible to certify that she is insane." No doubt this woman's mind is disordered. It is disordered to this extent, that her sexual craving is excessive; but even though she has this disorder of mind, she is not insane as long as she controls her conduct, and does not act in such a way as to satisfy her craving. Not until her conduct exhibits disorder can we say that she is mad; but as soon as she begins to act as a mad woman, so soon she is mad, and then it is not only justifiable, but imperative, to treat her as mad. We treat her as mad, not because she has this disorder in her mind, but because she gives expression to it in her behaviour.

